# Quantum Chemical and Experimental Evaluation of a 4-Amino-Antipyrine Based Schiff Base as Corrosion Inhibitor for Steel Material

**DOI:** 10.1021/acsomega.3c10048

**Published:** 2024-03-08

**Authors:** Ashwini Narayanswamy, Dileep Ramakrishna, P. V. Raja Shekar, Shashanka Rajendrachari, Ranganatha Sudhakar

**Affiliations:** † Department of Chemistry, Presidency University, Rajanakunte, Bengaluru 560064, India; ‡ Department of Physics, SR University, Warangal 506371, India; § Department of Metallurgical and Materials Engineering, Bartin University, Bartin 74100, Turkey

## Abstract

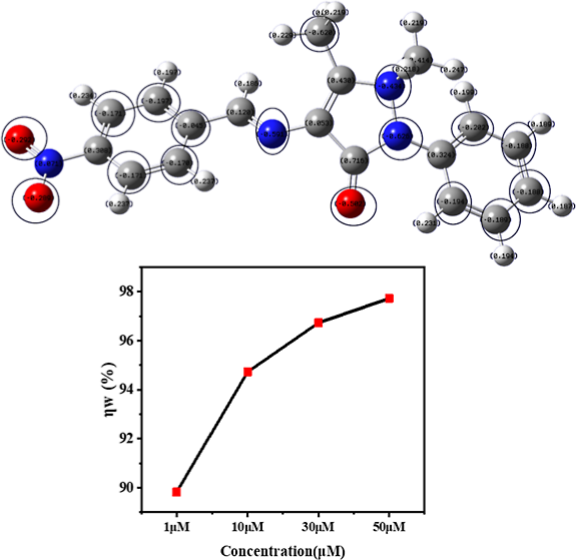

Electrochemical experiments such as potentiodynamic polarization, electrochemical impedance spectroscopy, and gravimetric studies have been used to examine the corrosion inhibitory efficacy of 4-[(4-nitrobenzylidene)-amino]-antipyrine (4-NBAAP) on mild steel (MS) in 1 M HCl. 4-NBAAP inhibits the corrosion of MS through a mixed inhibition mechanism, according to the electrochemical investigation. The efficiency of 4-NBAAP increases with an increase in the inhibitor concentration and decreases with an increase in temperature. The adsorption of 4-NBAAP molecules on the MS surface follows the Langmuir adsorption isotherm. To find the relationship between the 4-NBAAP molecular structure and inhibitive effect, a few thermodynamic parameters were computed. The experimental results obtained from gravimetric and different electrochemical investigations prove the superiority of the inhibitor at higher concentrations in controlling the corrosion process of the steel in aggressive environments. Also, quantum chemical studies were performed to provide further insights into the inhibition mechanism.

## 1 Introduction

The iron alloy of great industrial importance, mild steel (MS), is prone to get corroded in acidic environments due to thermodynamic instability. Despite the tendency to react readily with its environment, MS finds extensive applications in industrial establishments and machineries, as it is economically cheap and has superior mechanical properties. Keeping this in mind, the scientific community has developed various corrosion control strategies, and the one which is effective in reducing the corrosion rates is introducing inhibitor molecules to the aqueous corrosive media. Industrially applied inhibitors are mostly the organic molecules having hetero atoms that enhance their performance in protecting the metal surface from corrosion if the moiety possesses different functional groups, electron densities at donor atoms, and aromaticities. These kinds of features in the molecules support the adsorption on the MS surface to be protected.^1−4^

The condensation process between amines and carbonyl compounds produces Schiff bases, which have been shown to provide excellent corrosion prevention. Also, as the synthetic procedure is simple to carry out with cheap reactants, Schiff bases have become an attractive choice for industrial applications. These compounds are effective because of the presence of an electroactive imine group that facilitates easy adsorption on to the surface of a substrate metal. This effectiveness can be improved if the moiety possesses aromaticity/multiple bonds and participation of other hetero atoms/functional groups by facilitating electronic interaction with that of the d-orbital of Fe. Studies by various research groups suggest that the choice of Schiff bases as a corrosion inhibitor would be a successful strategy. The environment-friendly nature of the corresponding compounds is an added advantage to consider this for industrial applications.^5,15,17−19^

The Schiff bases synthesized from 4-amino-antipyrine (AAP) and suitable aldehydes have exhibited several functional properties such as antimicrobial, antioxidant, leishmanicidal, and corrosion inhibition due to their chemical structure and nature. There are very few detailed studies of AAP-based Schiff bases applied as a corrosion inhibition. Govindaraju et al. studied the synthesis and applications of AAP-based Schiff bases as corrosion inhibitors for MS in 1 M HCl.^1^ Different aldehydes were used to synthesize the Schiff base and evaluated using conventional gravimetric analysis and electrochemical experiment. Junaedi et al. employed a Schiff base produced from the composition of AAP and 4-nitrobenzaldehyde on MS in a 1 M Hydrochloric acid solution.^2^ The efficiency of the Schiff base obtained from AAP and substituted benzaldehydes was successfully evaluated by Eldesoky’s group in a 2 M HNO_3_ medium.^3^ The same molecules also exhibited an appreciable performance in a 2 M HCl solution.^4^ Balaji et al. proved the suitability of these Schiff bases prepared by composing AAP with different aldehydes such as 4-Nitrobenzaldehyde for MS in 1 M HCl.^5^ Transition-metal complexes of AAP-based Schiff bases also have performed as corrosion inhibitors along with biological activities.^6^ P110 steel, which is a medium carbon grade alloy steel, was studied in highly concentrated acid solution in the presence of AAP-based Schiff bases prepared with benzaldehyde and 4-nitrobenzaldehyde.^7^ Substituted benzaldehydes were used to prepare Schiff bases with AAP and evaluated for their inhibition efficiency in an acidic medium by Upadhyay et al.^8^ Abbass et al. studied the efficacy of 2-dimethylaminopropionamidoanti-pyrine in 1 M HCl to inhibit MS corrosion with the help of experimental and theoretical methods.^9^ A series of Schiff bases derived from some amino benzoic acid and AAP were synthesized by Okey and team, and their inhibition efficiency was tested in an aggressive 1 M HCl medium.^10^ Substituted benzaldehydes were composed of AAP, and the resulting compounds were evaluated for their inhibition mechanism by Madi et al.^11^ Raheef et al. prepared an AAP-based Schiff base using 2-aminothiozole and studied it in 1 M HCl for its efficiency and found appreciable inhibition.^12^ Hanoon and group conducted research on 4-((4-(dimethylamino)benzylidine)amino)antipyrine and found its appreciable inhibition capacity for MS material in 1 M HCl.^13^ A research group led by Aziz synthesized an AAP-based Schiff base composed of 2-methylbenzaldehyde and studied its inhibition efficiency in 1 M HCl for MS.^14^ Previous literature reports related to the Schiff base synthesized by composing AAP and 4-nitrobenzaldehyde were limited to either experimental studies or particular substrate material/corrosive medium.^4,8,11^ Application of this inhibitor molecule in a very aggressive and common industrially employed corrosive medium along with detailed experimental and theoretical evaluation is lacking in the literature. In this direction, the current contribution aims to evaluate corrosion inhibition efficiencies of a Schiff base synthesized using AAP and 4-nitrobenzaldehyde for MS material in 1 M hydrochloric acid solution.

## 2 Experimental Details

### 2.1 Chemicals and Materials

The heterocyclic compounds, 4-amino-antipyrine (C_11_H_13_N_3_O) and 4-nitrobenzaldehyde (C_7_H_5_NO_3_), were obtained from SRL Pvt. Ltd. Methanol (MeOH) was received from Merck. The above chemicals were used in Schiff base synthesis. FTIR analysis was performed utilizing the PerkinElmer Fourier transform infrared spectroscopy instrument. The Varian Mercury 400 MHz ^1^H NMR spectra were obtained through mass spectrum analyses using an Agilent 6224 HRMS spectrometer, with TMS serving as the internal standard. A Varian Mercury (VM) 100 MHz spectrometer was used to examine the ^13^C NMR data. ^13^C NMR chemical shifts (δ) were reported in ppm using the internal CDCl_3_ Μ 77.0 ppm.^15−17,20^

MS material was utilized to perform corrosion tests with the following compositions: 0.04 C, 0.022 P, 0.35 Mn, 0.036 S. MS specimens with dimensions of 0.1 × 3 × 3 cm were employed for gravimetric tests, whereas, for electrochemical experiments, a 1 cm^2^ exposed area and a 5 cm long stem were isolated using Araldite resin in the samples. MS samples were rubbed with emery papers of grades 220, 660, and 1200 as part of the pretreatment process. The samples were cleaned with double-distilled water and acetone, dried, and kept in a desiccator.^16−18,24,25^

### 2.2 Experimental Methods

#### 2.2.1 Synthesis

**Figure 1:**
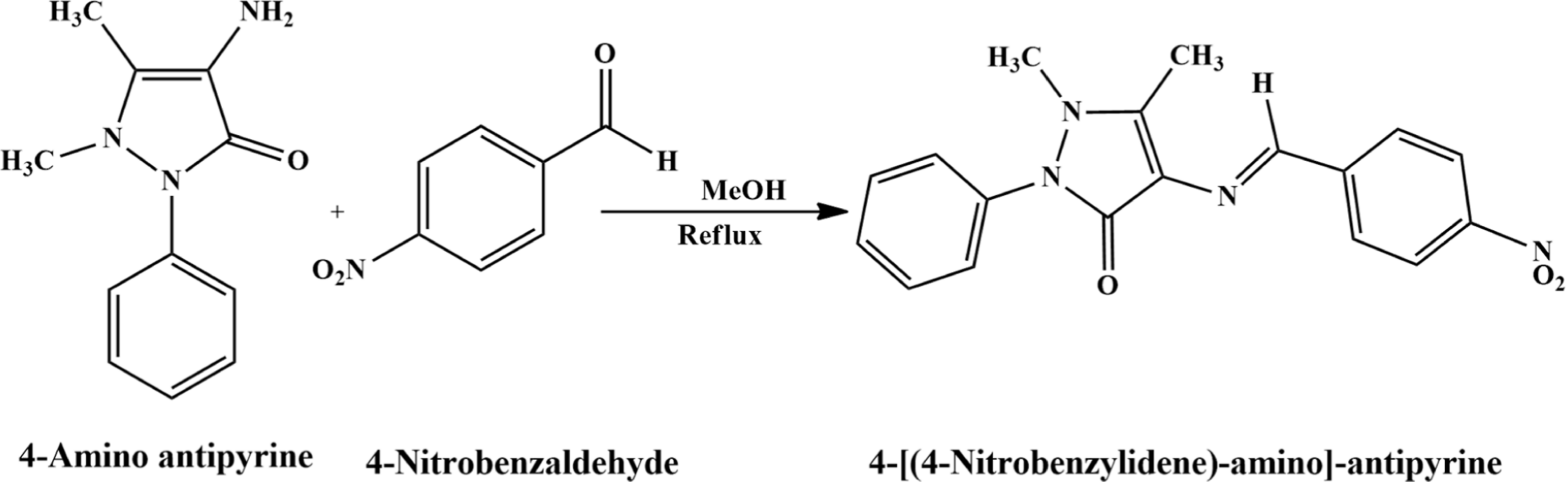
(Reaction scheme 1) molecular structure of 4-[(4-nitro benzylidene)-amino]-antipyrine (4-NBAAP).

The Schiff bases were synthesized and characterized by using physicochemical methods. The synthetic route for the synthesis of the Schiff base is given in Scheme 1. The Schiff base, 4-[(4-nitro benzylidene)-amino]-antipyrine (4-AAPNB), was prepared (as shown in Reaction Scheme 1) by an equimolar mixture (1:1) of methanolic medium of 4-amino-antipyrine (0.2000 g) and 4-nitrobenzaldehyde (0.122 g) in a round-bottom flask. In addition, 30 mL of methanol (MeOH) was added followed by a 4 h reflux at 303 K. An orange/yellowish colored precipitate thus obtained was purified and filtered through two to three methanol washes to eliminate any remaining reactants and then vacuum-dried. Analytical grade chemicals and double-distilled water were used for the entire experiment. Molecules thus prepared were utilized for further studies (Figure 1).^15−19,57,58^

#### 2.2.2 Characterization of 4-[(4-Nitrobenzylidene)-amino]-antipyrine

*FTIR, 1H NMR, 13CNMR, and Mass Spectral Analysis*.

Yield: 0.61 g (81%),

Melting point: 116–120 °C.

Solubility: soluble in polar solvents such as water, methanol, ethanol, ethyl acetate, acetonitrile, non-polar solvent toluene.

Anal. Calcd for C_17_H_16_N_4_O_3_ (336.12): C, 64.28; H, 4.79; N, 16.66; O, 14.27.

Elemental analysis (CHN): C, 64.28; (100.0%) H, 4.79; (19.8%) N, 16.66; (2.5%) O, 14.27.

FTIR: 3047.4 ν (aromatic C–H), 16,702.1 ν(C=O), 1526.3 ν(C=N).

^1^H NMR of 4-[(4-nitro benzylidene)-amino]-antipyrine shows the presence of peaks 9.505 (s,1H), 8.423 (s,1H), 7.484–7.527 (m,4H), 7.601–7.621 (d,3H), 7.226 (s,1H), 6.869–6.889 (d,3H), 3.728 (s,3H). ^13^C NMR 160.776, 158.498, 157.706, 145.545, 143.114, 140.882, 130.824, 129.498, 125.490, 122.648, 117.688, 114.144, 104.810, 99.012, 55.428.

Mass: 337.06 *m*/*z* (100%).^19−24^ (Figure S1)

#### 2.2.3 Medium

Analytical reagent grade 37% hydrochloric acid was diluted to prepare 1 M HCl using double-distilled water. Acidic solutions for corrosion study were prepared by dissolving a specific amount of corrosion inhibitor molecules in 1 M HCl.^24−27^

#### 2.2.4 Inhibitor Solutions

The desired concentrations of inhibitor solutions (10, 30, and 50 μM) were prepared by dissolving a specified amount of 4-[(4-nitro benzylidene)-amino]-antipyrine (4-NBAAP) in 1 M HCl solutions.^23,24^

### 2.3 Gravimetric Analysis

Different MS strips were immersed in 1 M HCl media having different concentrations of the 4-NBAAP inhibitor for about 4 h at room temperature. The weight differences of MS strips without and with the inhibitor were recorded and used to calculate the inhibition efficiency (IE %).^25−28^

#### 2.3.1 Electrochemical Analysis

The electrochemical experiments, including a three-electrode, were adopted using a CHI 608C electrochemical analyzer (CH Instruments, 3700 Tennison Hill Drive, Austin, TX 78738). The working electrode was the MS sample with a 1 cm^2^ exposed area and Sat. Calomel and Pt were reference and auxiliary electrodes, respectively. Samples were allowed to get stabilized over a period of 30 min for their open circuit potential (OCP) before every electrochemical experiment.^25−29^

#### 2.3.2 Potentiodynamic and Polarization Studies

The potentiodynamic polarization studies were performed over a potential from +200 to −200 mV at the OCP with a scan rate of 0.5 mV s^–1^. Corrosion characteristics such as corrosion potential (*E*_corr_), corrosion current (*I*_corr_), and anodic (β_a_)/cathodic (β_c_) Tafel slopes were obtained from the installed software.

The following relation was used to calculate the inhibition efficiencies (IE %) based on the obtained *I*_corr_ values.^27,30^
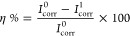
1where *I*_corr_^0^ and *I*_corr_ are the corrosion current densities without and with 4-NBAAP, respectively.

#### 2.3.3 Electrochemical Impedance Spectroscopy

The electrochemical impedance spectroscopy (EIS) measurements were carried out in the frequency range of 1–10 MHz at corresponding OCP with an excitation signal of 5 mV. The obtained impedance results were analyzed with ZSimp-Win 3.21 software.^33−35^

With the help of the following equation, the inhibition efficiency was evaluated.
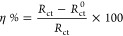
2where *R*_ct_ and *I*_ct_^0^ are the charge transfer resistance with and without 4-NBAAP, respectively.

Potential values reported were with reference to the Sat. Calomel electrode. The reported values were the mean of triplicate measurements.^27−35^

### 2.4 Morphological Studies

A scanning electron microscope Tescan Vega 3 was used to study morphological changes with corrosion in the presence and absence of the tested inhibitor in a corrosive medium. The working distance was 15 mm with an electron voltage of 15 kV and a magnification of 1000×, and a secondary electron detector was used to capture the images.

### 2.5 Quantum Chemical Studies

Quantum chemical calculations were carried out by using Gaussian 09 software and its time-dependent extension TDDFT at the CAM-B3LYP/6-31G level to ascertain the correlation between the molecular and electronic structure and inhibition efficiency of 4-NBAAP.

## 3 Results and Discussion

### 3.1 Structural Analysis

Results are provided in the Supporting Information.

#### 3.1.1 UV–Vis Analysis

The bands in the region of 265–280 nm are assigned to the π–π* transitions of the aromatic rings. The band at 309–357 nm involves an *n*–π* transition of the C=O and C=N groups. The longer wavelength band at 390–402 nm can be assigned to an intramolecular charge transfer transition. The charge transfer will originate from a 4-aminoantipyrine ring as an origin to the C=N group as a sink.^20,21,29^ (Figure S2)

#### 3.1.2 FT-IR Spectral Studies

The FT-IR spectra of the ligands exhibit intense bands at 1635–1650 and 1610–1620 cm^–1^ corresponding to antipyrine exocyclic ketone ν(C=O) and azomethine ν(CH=N), respectively. The band in the region of 1285–1310 cm^–1^ is assigned to phenolic ν(C–O). Thus, a ligand is tridentate in nature with O, N, O coordination sites. In the spectrum, ν(CH=N), i.e., around 1600 cm^–1^ confirms the presence of azomethine nitrogen.^22,23,30,31,57^ (Figure S3)

#### 3.1.3 NMR Spectroscopy

The ^1^H NMR spectra of the ligands (Figures S4 and S5) display two sharp signals (3H) at δ 2.4 and δ 3.10–3.19 ppm corresponding to the (=C–CH_3_) and N–CH_3_ groups, respectively. A singlet (1H) observed at 9.7–10.6 ppm in the spectra of ligands has been assigned to an azomethine proton (−CH=N). A sharp singlet (1H) appeared for the o–OH proton of the ligand in the region δ 12–14 ppm.^21,22^ In the ^13^C NMR spectra of the molecule, the azomethine carbon resonance is observed at the 154 ppm range. The resonances for C–N and C–O are observed in the regions 145 and 160 ppm, respectively. The ^13^C NMR spectra of complexes revealed the presence of six different carbons (119, 129, 143, 143, 149, and 159 ppm). The three quaternary carbons arising from aromatic units are in the same magnetic environments.

### 3.2 Gravimetric Analysis

The 100 cm^3^ of 1 M hydrochloric acid was taken in beakers for the purpose of measuring weight loss. After the first beaker was fixed as an uninhibited blank solution, the remaining five beakers were filled with 1, 10, 30, and 50 μM concentrations of the inhibitor. In each of the five inhibited solutions, polished and pretreated MS strips were immersed for approximately 4 h (298 K). We measured the weight variations of each strip both before and after they were immersed in the solution (Figure S6). The corrosion rate for each strip is provided by the following equation
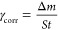
3where Δ*m* is the corrosion weight loss of the MS (mg), *S* is the surface area of the MS sample (cm^2^), and *t* is the time of exposure.

The percentage inhibition efficiency η_w_ (%) was calculated using the relationship
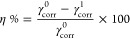
4where γ_corr_^0^ and γ_corr_^1^ are the corrosion rates (CR) of the MS in the absence and presence of 4-[(4-nitrobenzylidene)-amino]-antipyrine, respectively (Table 1).

**Table 1:** Corrosion Parameters Obtained by the Weight Loss Measurement in 1 M Hydrochloric Acid at Room Temperature at 298 K

	concentration	*W*_0_	*W*_1_	*W*_(g)_	θ	η %	CR (mgcm^–2^ h^–1^)
4 h	blank	1.576	1.466	0.11	0.0698		1.2 × 10^–^^5^
	1 μM	1.6568	1.631	0.0258	0.01557	76.54	2.9 × 10^–^^6^
	10 μM	1.6977	1.6722	0.0255	0.01502	76.81	2.8 × 10^–^^6^
	30 μM	1.6279	1.6073	0.0206	0.01265	81.27	2.3 × 10^–^^6^
	50 μM	1.7214	1.704	0.0174	0.01011	84.18	1.9 × 10^–^^6^

#### 3.2.1 Effect of Temperature

**Figure 2:**
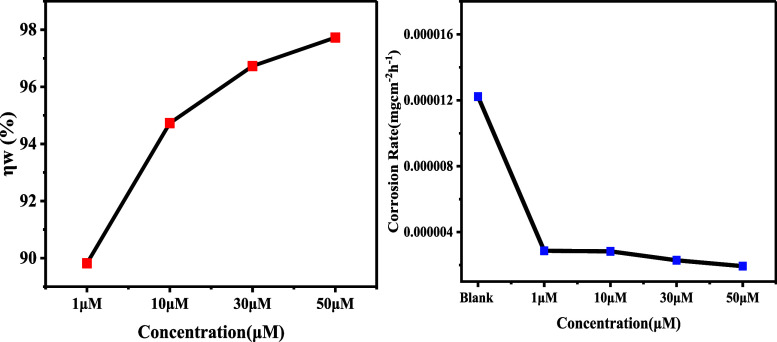
Corrosion efficiency and corrosion rate variation with different inhibitor concentrations.

**Figure 3:**
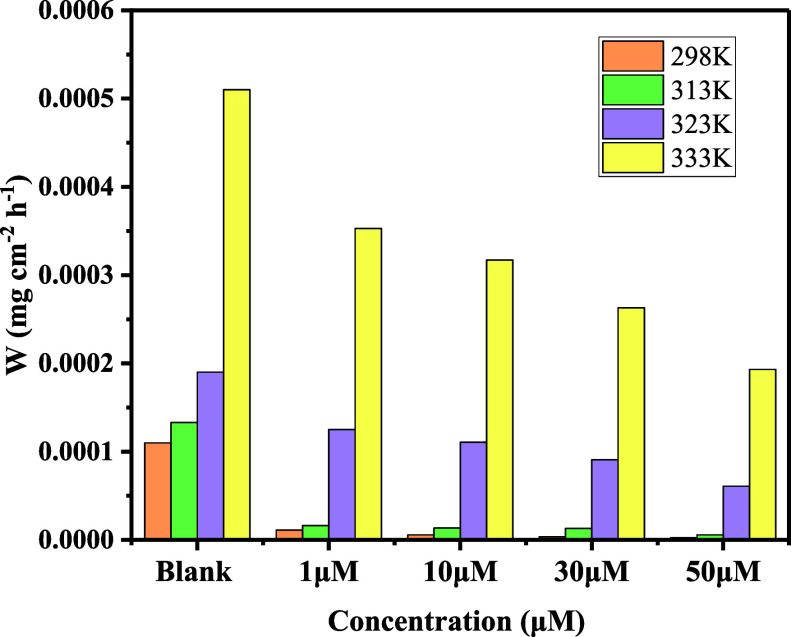
Effect of temperature on weight loss in 1 M HCl at different concentrations of the inhibitor (Figure S6).

We examined weight loss measurements in the 298–333 K temperature range both with and without 4-NBAAP in a 1 M HCl solution to ascertain how temperature affects the process of corrosion inhibition. Table 2 provides the basis for the corrosion parameters that are derived from weight loss measures. An increase in temperature rates upward of the evolution of hydrogen gas increases the rate at which MS oxidizes. As a result, as the temperature increases, the amount of inhibitor adsorption decreased and the MS dissolved more quickly.^26−35^ In the current investigation, corrosion rates rise and inhibition efficiency falls in 1 M HCl media at constant concentrations of 4-NBAAP. Higher temperatures do not support the adsorption of inhibitor molecules, which is the reason behind this observation (Figures 2 and 3).

**Table 2:** Corrosion Parameters at Different Temperatures^a^

*C* (mM)	temperature											
	298 K			313 K			323 K			333 K		
	CR (mg cm^–2^ h^–1^)	η_w_ (%)	Θ	CR (mg cm^–2^ h^–1^)	η_w_ (%)	θ	CR (mg cm^–2^ h^–1^)	η_w_ (%)	Θ	CR (mg cm^–2^ h^–1^)	η_w_ (%)	θ
blank	1.2 × 10^–^^5^			1.5 × 10^–^^5^			2.1 × 10^–^^5^			5.7 × 10^–^^5^		
1 μM	1.2 × 10^–^^6^	89.81	0.89818	1.81 × 10^–^^6^	87.74	0.87744	1.4 × 10^–^^5^	34.15	0.34157	3.9 × 10^–^^5^	30.80	0.3080
10 μM	6.4 × 10^–^^7^	94.72	0.94727	1.51 × 10^–^^6^	89.77	0.89774	1.2 × 10^–^^5^	41.73	0.41736	3.5 × 10^–^^5^	37.82	0.3782
30 μM	4 × 10^–^^7^	96.72	0.96727	1.46 × 10^–^^6^	90.15	0.9015	1 × 10^–^^5^	52.10	0.52105	2.9 × 10^–^^5^	48.43	0.4843
50 μM	2.8 × 10^–^^7^	97.72	0.97727	6.33 × 10^–^^7^	95.71	0.95714	6.8 × 10^–^^6^	67.94	0.67947	2.1 × 10^–^^5^	62.13	0.6213

a Figure S6.

#### 3.2.2 Adsorption Isotherm and Thermodynamic Consideration

**Figure 4:**
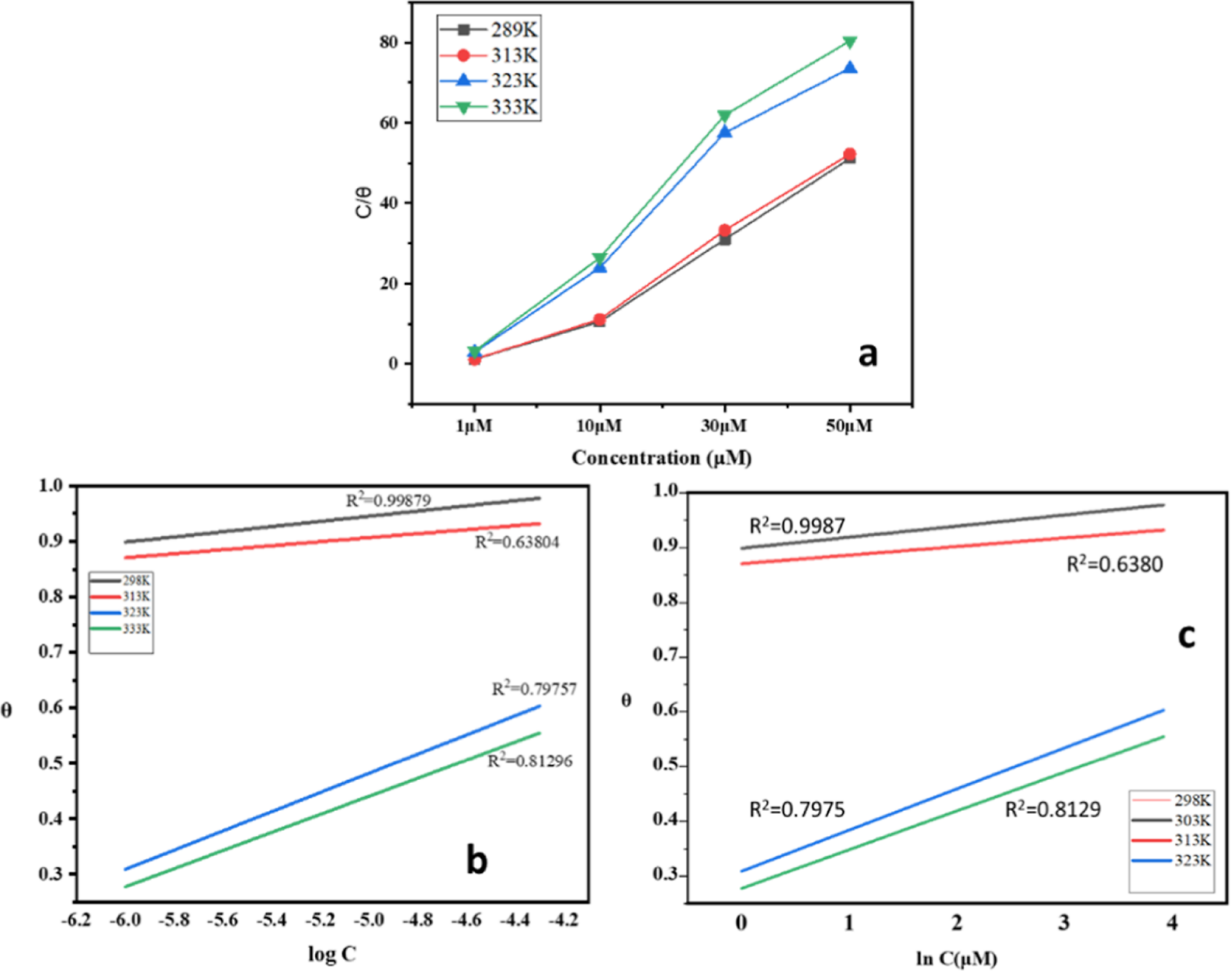
(a) Langmuir (b) Temkin, and (c) Freundlich adsorption isotherm plots of 4-NBAAP in a 1 M HCl solution on the MS surface.

Figure 4 depicts the adsorption isotherm concerning the way in which inhibitor molecules interact with the metal surface. Among different adsorption isotherm models that we fitted to the experimental data, the Langmuir isotherm was found to be the best fit. Figure 4, also shows few isotherm systems as an example, which are not fitting well with the experimental values, showing lower values for regression coefficients. Adsorption of 4-NBAAP on MS surfaces in hydrochloric acid follows the Langmuir adsorption isotherm, which can be mathematically expressed as follows.
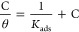
5where *C* is the inhibitor concentration, θ is the degree of the surface coverage, and *K*_ads_ is the adsorption equilibrium constant.

**Figure 5:**
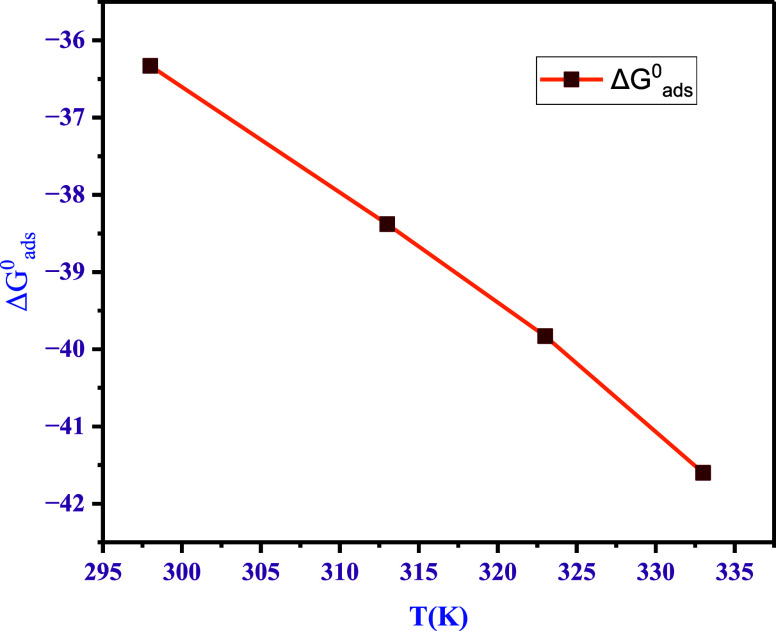
Relation between temperature (*T*) and Δ*G*_ads_^0^ (kJ mol^–1^).

The Langmuir adsorption isotherm plots and the corresponding *C*/θ vs *C* plots obtained for 4-NBAAP are linear with a nearly unit slope, which are presented in Figures 4 and 5. The standard free energy of adsorption (Δ*G*_ads_^0^) and the *K*_ads_ can be determined using the intercepts of the straight lines on the *C*/θ axis.^26^
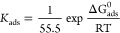
6where *R* is the universal gas constant, *T* is the thermodynamic temperature, and 55.5 is the molar concentration of water in the media.

The adsorption parameters are shown in Table 3. The stability of the adsorbed layer on the MS surface in 1 M hydrochloric acid media and the spontaneity of the adsorption process can be related to the large negative values of Δ*G*_ads_^0^.^33^ In general terms, physisorption is associated with Δ*G*_ads_^0^ values of −20 kJ mol^–1^ or less, and chemisorption is indicated by values of −40 kJ mol^–1^ or more.^34^ However, in 1 M HCl solutions, the calculated Δ*G*_ads_^0^ values are between −40 and −20 kJ mol^–1^. Both chemisorption and physisorption are involved in the adsorption of 4-NBAAP on a MS.

**Table 3:** Thermodynamic Parameters of the Adsorption of 4-NBAAP on the MS Surface at Different Temperatures

temperature (K)	*R*^2^	*K*_ads_ (M^–1^)	Δ*G*_ads_^0^ (kJ mol^–^^1^)	Δ*H*_ads_^0^ (kJ mol^–^^1^)	Δ*S*_ads_^0^ (J mol^–^^1^ K^–^^1^)
298	0.9866	42,070	–36.33		–122.1
313	0.9831	45,690	–38.38	–72.56	–122.4
323	0.9614	49,700	–39.83		–123.1
333	0.9797	51,500	–41.6		–123.4

In general, as the temperature increases, Δ*G*_ads_^0^ increases (become less negative), signifying an exothermic process; and decrease in Δ*G*_ads_^0^ (becomes more negative) indicates the process to be an endothermic one. Table 3 makes it evident that as temperature rises, Δ*G*_ads_^0^ rises as well, indicating that 4-NBAAP corrosion inhibition of MS is an exothermic process. Here, adsorption of 4-NBAAP on the metal surface becomes unfavorable as the reaction temperature rises.^35−45^ The integrated version of the Van’t Hoff equation can be used to calculate the entropy of adsorption and the enthalpy of adsorption (Δ*H*_ads_^0^) and it is expressed as follows.^46−54^
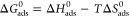
7Figure 5 shows a linear plot of Δ*G*_ads_^0^ against *T*, where the intercept is equal to Δ*H*_ads_^0^ and the slope is equal to Δ*S*_ads_^0^. The values of Δ*S*_ads_^0^ and Δ*H*_ads_^0^ that were obtained are −123.4 and −72.56 kJ mol^–1^, respectively.

The inhibitor adsorption on MS exhibits an exothermic behavior, as indicated by the negative values of Δ*H*_ads_^0^. An exothermic adsorption process typically denotes chemisorption or physisorption.^55^ When examining the absolute value of Δ*H*_ads_^0^, one can differentiate between physisorption and chemisorption in an exothermic process. In the case where Δ*H*_ads_^0^ is lower than that of 40 kJ mol^–1^, it is considered to be physisorption, while that for chemisorption approaches the value approaching 100 kJ mol^–1^.^44^ In the present case, Δ*H*_ads_^0^ is −72.56 kJ mol^–1^; the existence of both physical and chemical adsorption is clearly observed in this intermediate case, and thus Δ*G*_ads_^0^ and Δ*H*_ads_^0^ values complement each other. As expected, the negative values of Δ*S*_ads_^0^ pronounce the decrease in entropy with respect to an exothermic adsorption process. In a bulk solution, the inhibitor molecules are free to migrate and as the adsorption process proceeds, the inhibitor molecules’ adsorption onto the MS surface becomes more organized, which lowers entropy.^46−56^ Moreover, the values of two methods’ results for Δ*H*_ads_^0^ and −Δ*S*_ads_^0^ are in good agreement.

### 3.3 Electrochemical Studies

#### 3.3.1 OCP Measurements

**Figure 6:**
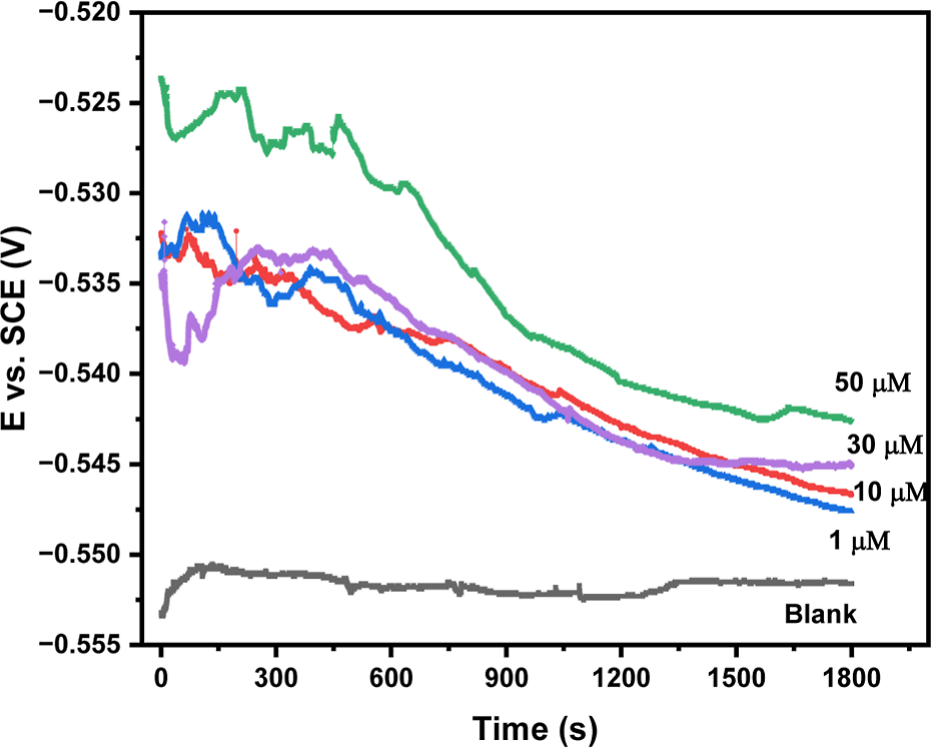
OCP profiles of the samples at 298 K with and without 4-NBAAP in 1 M HCl.

OCP measurements were performed prior to the other electrochemical experiments. OCP signifies the equilibrium potential in the open circuit with no electrical interruption or without applying any external load. This equilibrium forms between the electrode surface and electrolyte interface, and it deviates in case of any change in the interface or reactions on the surface of the electrode.^21^Figure 6 presents OCP profiles for the steel specimens under different environments measured for 1800 s. The sample in 1 M HCl with no inhibitor attains −0.55 V, and the samples with different inhibitor concentrations attain potentials with least negative values, i.e., −0.542 V in the case of 50 μM. This proves that a higher concentration of the inhibitor facilitates the electrochemical stability of the steel specimen.

##### 3.3.1.1 Potentiodynamic Polarization Studies

**Figure 7:**
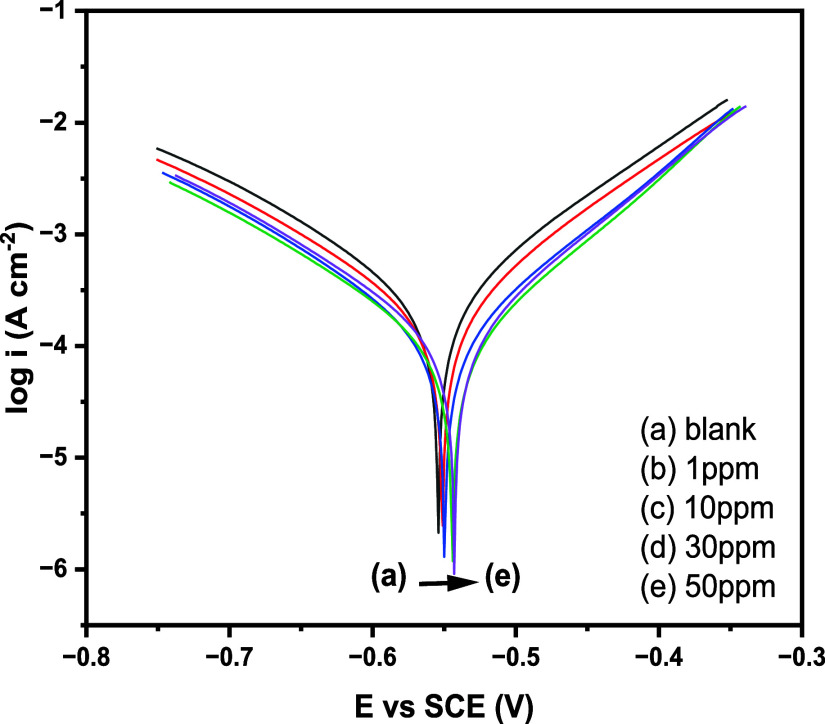
Plots of Tafel polarization for the samples at 298 K with and without 4-NBAAP in 1 M HCl.

The potentiodynamic polarization curves of the samples in 1 M hydrochloric acid in the presence and absence of 4-NBAAP are shown in Figure 7. Table 4 shows the corrosion kinetic parameters derived from these curves. Both the anodic and cathodic branches of the polarization curves of the acid solutions shifted toward a lower current density at all investigated concentrations in acid media, as the figure illustrates. This suggests that 4-NBAAP in the 1 M HCl solution inhibited both the anodic and cathodic reactions of MS corrosion. As inhibitor concentration rises, protection efficiency rises and corrosion current density falls. The addition of the inhibitor resulted in a positive shift in the *E*_corr_ values, and the values of β_c_ and β_a_ did not significantly change. Also, if and only if the *E*_corr_ shift is more than 85 mV, the inhibitor can be designated as either cathodic or anodic in nature. This implies that 4-NBAAP functions as an inhibitor of a mixed type.^46,47^

**Table 4:** Potentiodynamic Polarization Parameters with and without Varying the 4-NBAAP Concentrations in 1 M HCl

*C* (μM)	–*E*_corr_ (mV)	–*b*_c_ (V^–1^)	–*b*_a_ (V^–1^)	*I*_corr_ (μA cm–^2^)	standard deviation	η_T_ (%)
blank	483.7	9.282	11.271	270 ± 0.9	0.9	
1 μM	549.0	8.032	9.358	131 ± 1.2	1.2	51.48
10 μM	550.2	8.427	10.144	115 ± 2.3	2.3	57.40
30 μM	541.2	8.531	10.735	105 ± 2.1	2.1	61.11
50 μM	545.0	8.149	10.504	94 ± 1.5	1.5	65.18

The inhibition efficiency (eq 8) is enhanced as a result of the inhibitor molecule’s increasing surface coverage (caused by an increase in inhibitor concentration). A maximum of 65% inhibition efficiency is seen at 298 K, which is corresponding to the inhibitor concentration of 50 μM.
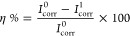
8

#### 3.3.2 Electrochemical Impedance Spectroscopy

**Figure 8:**
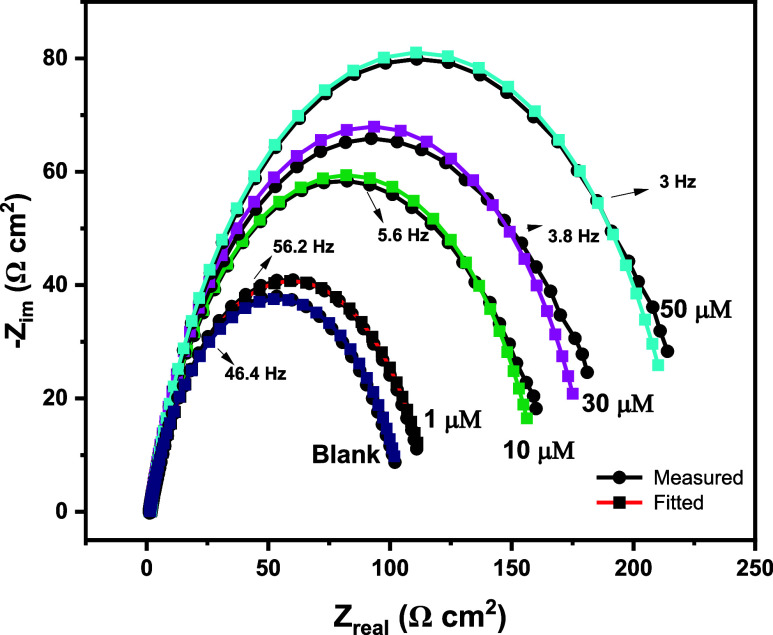
Nyquist plots of MS in 1.0 M hydrochloric acid without and with different concentrations of 4-NBAAP.

**Figure 9:**
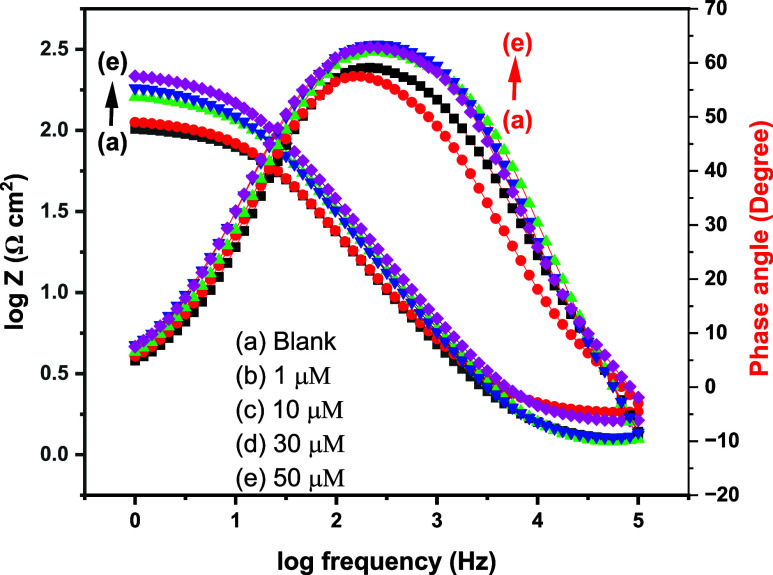
MS Bode plots in a 1 M HCl solution with and without varying the concentration of 4-NBAAP.

**Figure 10:**
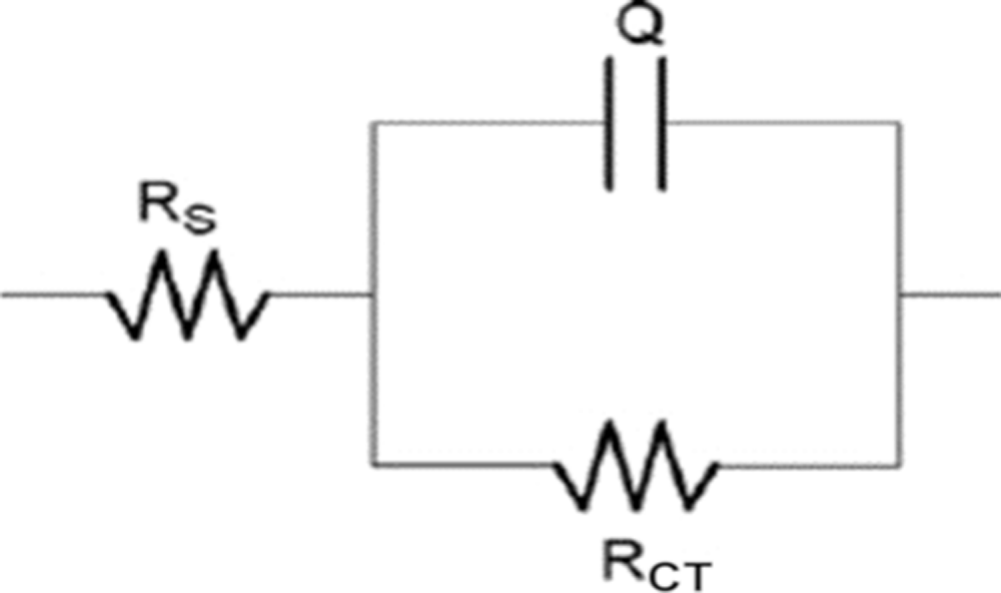
Electrochemical equivalent circuit model used to fit the impedance data. *R*_s_ = solution resistance, *R*_ct_ = charge transfer resistance, and *Q* = CPE.

Electrochemical impedance spectroscopy was used to assess MS corrosion behavior in 1 M hydrochloric acid with and without 4-NBAAP. The typical impedance data as Nyquist plots are shown in Figure 8 and Bode plots in Figure 9, and the corresponding parameters are presented in Table 5 with the equivalent circuit considered shown in Figure 10

**Table 5:** Electrochemical Impedance Parameters of MS in 1 M Hydrochloric Acid Containing Different Inhibitor Concentrations

*C* (μM)	*R*_S_ (Ω^–1^ cm^–2^)	*R*_ct_ (Ω cm^–2^)	standard deviation	*Q* (μΩ^–1^ Sn cm^–2^)	*n*	*C*_dl_ (μF cm^–2^)	χ^2^ × 10^–3^	η_Z_ (%)
blank	1.25	104.9 ± 1.2	0.96	250	0.79	4.7461	3.05	
1	1.79	115.3 ± 1.6	1.6	240	0.78	4.2924	1.04	31.19
10	1.17	162.3 ± 1.3	1.69	160	0.80	3.5565	4.96	35.36
30	1.23	182.9 ± 0.9	0.9	160	0.81	3.2884	3.83	42.64
50	1.55	220.3 ± 0.8	0.8	140	0.81	3.1870	1.08	52.65

These graphs show that the addition of 4-NBAAP to 1 M hydrochloric acid dramatically altered the impedance response of the MS. Additionally, the impedance spectra are composed of a single capacitive loop, and as the inhibitor concentration rises, so does the diameter of the loops. This suggests that 4-NBAAP functions as the main interface inhibitor in hydrochloric acid media and that inhibitor adsorption happens via simple surface coverage.^46^ The similar nature of Nyquist loops indicates that the mechanism of corrosion involved in the presence and absence of the inhibitor remains the same. For a more precise fit, a constant phase element (CPE) is used in place of the capacitive element (*C*_dl_). The electrochemical equivalent circuit used to analyze the impedance data is shown in Figure 10. The fitting standard of the equivalent circuit was checked by the chi-square (χ^2^) values. Generally, the values of χ^2^ among 10^–3^ and 10^–5^ suggest an ideal fit.^48^ The proposed equivalent circuit provided fairly small (<5 × 10^–3^) χ^2^ values, suggesting a satisfactory fitting of the obtained impedance spectra to the proposed equivalent circuit.
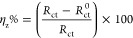


The CPE impedance (*Z*) can be expressed as
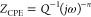
9where *Q* is the CPE, ω is the angular frequency, *j*^2^ is −1, *n* is the CPE exponent, and *C*_dl_ is the double-layer capacitance.^49^

Using the following formula, *C*_dl_ values can be obtained
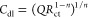
10

As the inhibitor concentration in hydrochloric acids increases, it is clear from the table that *Q* falls and *R*_ct_ increases. A protective layer forming on the electrode surface is the reason for the increase in *R*_ct._(44) By contrast, a decrease in the local dielectric constant and/or an increase in the electrical double layer’s thickness could have caused the *C*_dl_ to drop.^45−56^ With a higher concentration of hydrochloric acids, 4-NBAAP’s protection efficacy rises. As observed in the Bode plots, the increase in mod Z and phase angle shift with increased inhibitor concentration are attributed to the better inhibition performance. Here, increased inhibitor concentration results in progress in film formation and surface coverage, which is reflected in the shift and increase in the phase angle values. “*n*” indicates the surface roughness or surface inhomogeneity, and a slight variation in its value can be ascribed to minimization of surface inhomogeneity due to the adsorption of inhibitor molecules on the active adsorption sites.^44−56^ According to these findings, 4-NBAAP inhibits MS corrosion effectively in aggressive HCl solution, and it works wonderfully as an MS corrosion inhibitor. Potentiodynamic polarization curves and EIS-obtained inhibition efficiencies agree fairly well.

While our results were compared with the literature reports, the following observations were made. Govindaraju et al. studied the synthesis and applications of AAP-based Schiff bases obtained from different aldehydes and AAP, and the maximum inhibition efficiency obtained was 90.83% at 0.008 M.^1^ 4-Nitrobenzaldehyde and AAP Schiff base studied by Junaedi et al.^2^ shows an efficiency of 87% at 0.5 mM in 1 M HCl solution. Eldesoky’s group, which produced Schiff bases from substituted benzaldehydes, showed their maximum efficiency in the range of 41–48% in 2 M HCl.^4^ 2-Dimethylaminopropionamidoanti-pyrine exhibited a highest inhibition of 91.9% at a 5 mM concentration of the inhibitor in 1 M HCl.^9^ Experimental results obtained for the Schiff base obtained from amino benzoic acid and AAP show the corrosion inhibition to be up to 97% at a 1 mM inhibitor concentration.^10^ The efficiency of Schiff bases designed from substituted benzaldehydes and AAP claimed to be efficient up to 95.03% at a 0.0005 M inhibition concentration according to the experiments performed.^11^ The compound prepared out of 2-aminothiozole and AAP showed 88% efficacy at a 500 ppm concentration.^12^ 95% efficiency toward corrosion inhibition was reported in the case of 4-((4-(dimethylamino)benzylidine)amino)antipyrine at 0.5 mM^13^*N*-2-methylbenzylidine-4-antipyrineamine, 91.8% at 5 × 10^–4^ M.^14^

### 3.4 Surface Morphological Study

**Figure 11:**
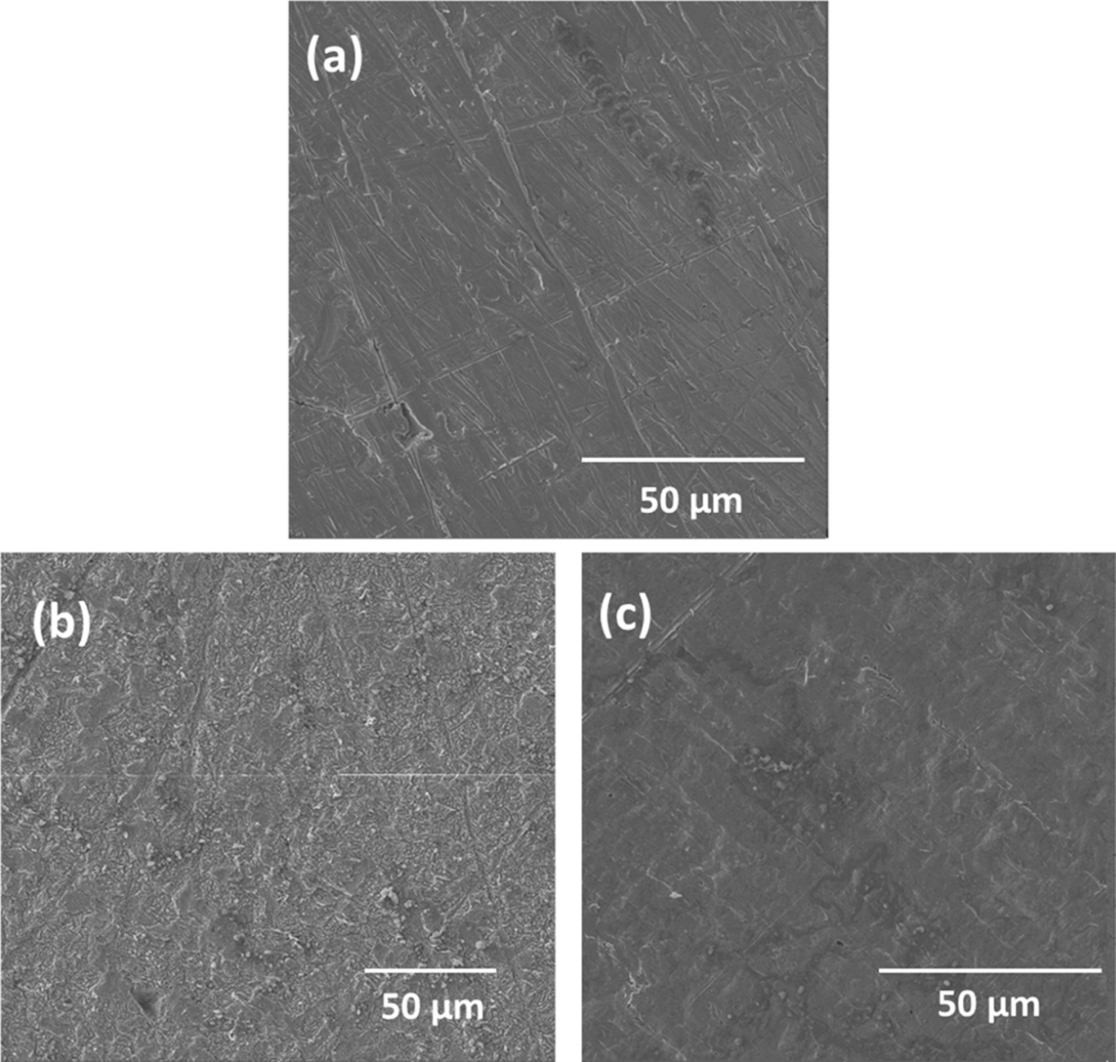
SEM images of the metal samples at different conditions: (a) bare and (b) after immersion for 4 h in 1 M HCl without the inhibitor and (c) with the inhibitor.

To establish the corrosion inhibition of 4-NBAAP, morphological studies were carried out and SEM images of the metal surfaces were analyzed. Figure 11a–c represents the surface morphology of the metal surfaces in different conditions such as in the bare condition and after 4 h of immersion in an uninhibited solution and an inhibited solution, respectively. In the absence of the inhibitor, the steel sample degraded significantly, and rust formation was visible. However, in the presence of the inhibitor, the rate of rust formation was diminished, lessening the metal degradation and proving the inhibition capacity of 4-NBAAP in an aggressive corrosive environment.

### 3.5 Quantum Chemical Calculations

**Figure 12:**
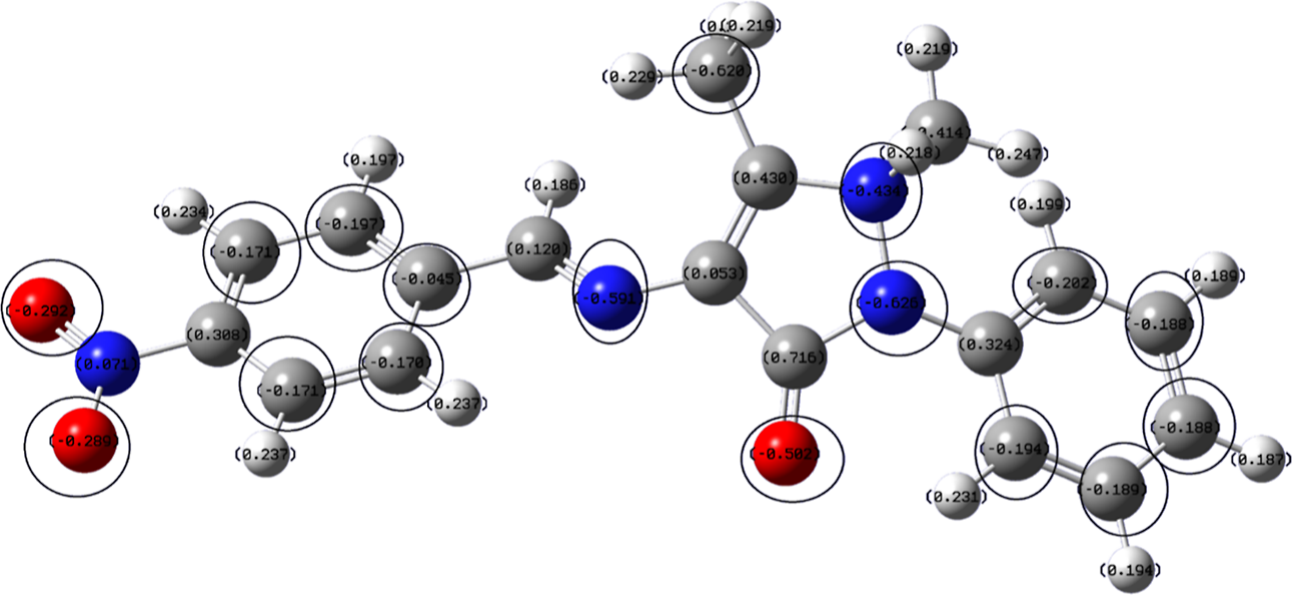
Optimized structure with a Mulliken charge distribution (negative charges encircled) of the 4-NBAAP molecule.

**Figure 13:**
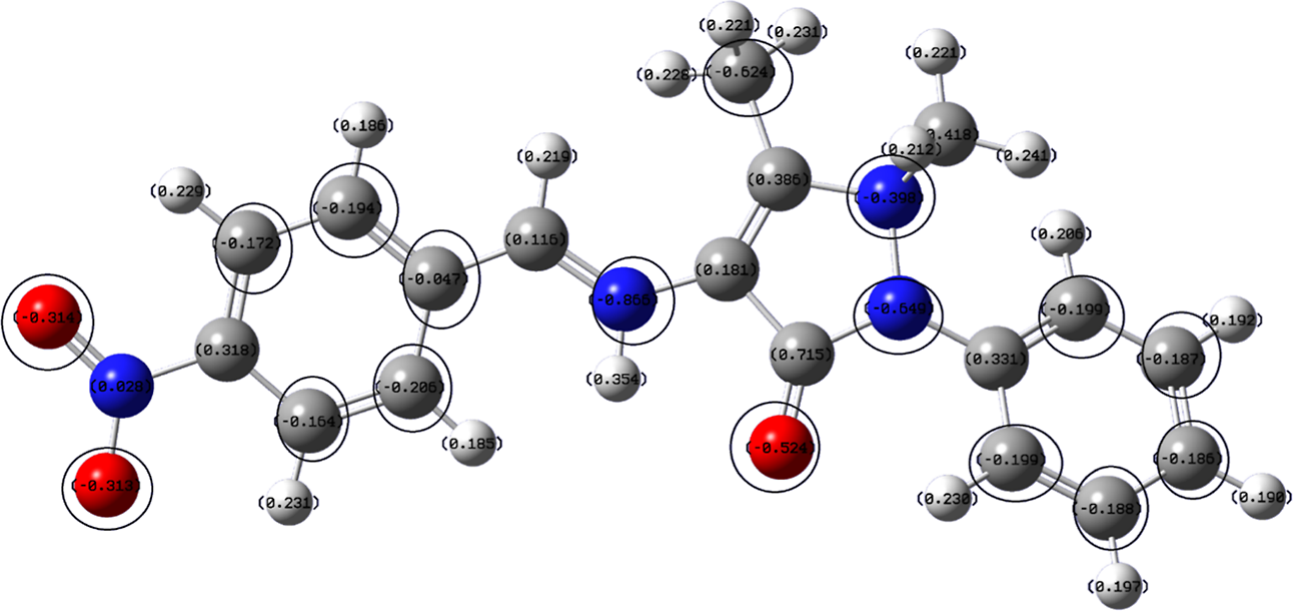
Optimized structure with a Mulliken charge distribution (negative charges encircled) of the protonated species of the 4-NBAAP molecule.

**Figure 14:**
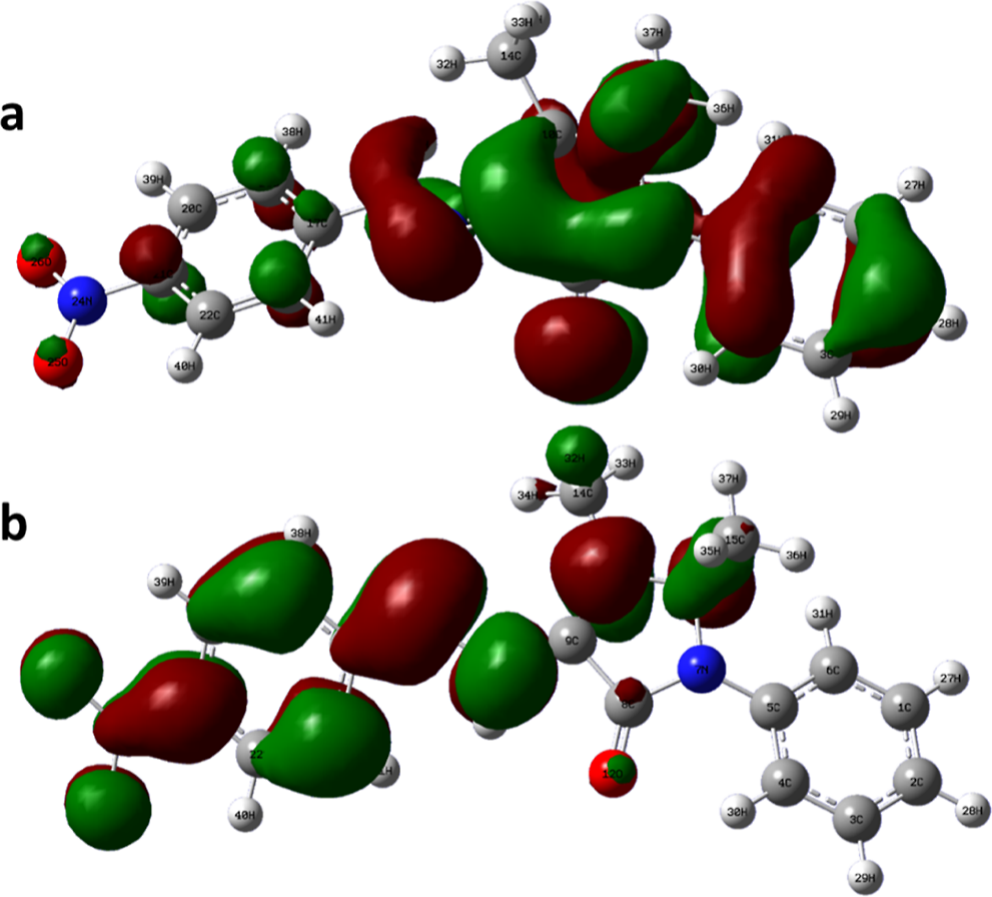
Frontier orbitals (HOMO) of (a) neutral and (b) protonated species of the 4-NBAAP molecule.

**Figure 15:**
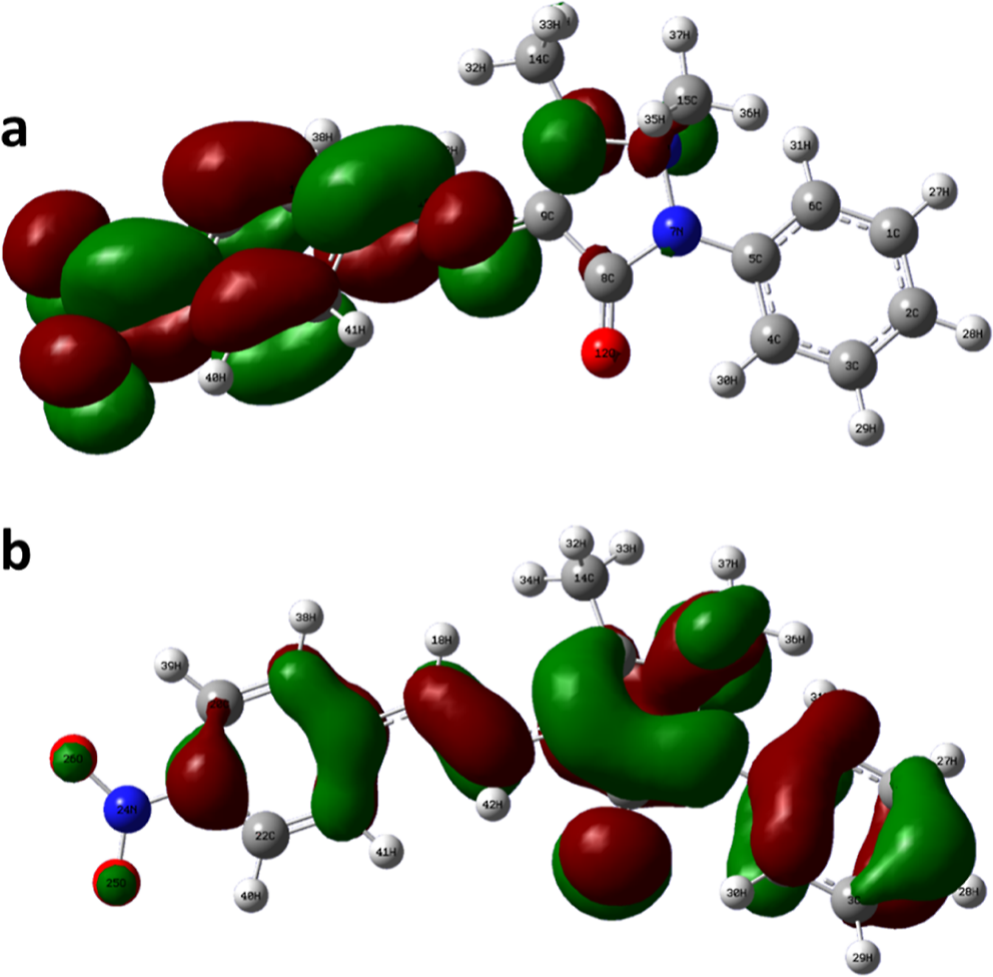
LUMO of the (a) neutral and (b) protonated species of the 4-NBAAP molecule.

The Gaussian software was used to optimize the structure of the inhibitor (Berny optimization using GEDIIS/GDIIS optimizer), and the optimized structures of both nonprotonated and protonated species along with active centers are presented in Figures 12 and 13. correspondingly. The distributions of the highest occupied molecular orbitals (HOMO) and lowest unoccupied molecular orbitals (LUMO) are shown in Figures 14 and 15, respectively. The Mulliken population is a good tool to probe adsorption centers present in an inhibitor molecule; these are negatively charged atoms in the molecule, and the same are highlighted in Figures 12 and 13. The general consensus is that adsorption centers are the negatively charged heteroatoms and negatively charged carbon atoms of an aromatic ring. The more the magnitude and the number of negative charges present in a molecule, the more it is adsorbed on to the metal surface. The total negative charge (TNC) value would be an important parameter to quantify the adsorption centers present in a molecule. A higher TNC value indicates a larger charge separation and hence the presence of good adsorption sites. The TNC for the neutral and protonated inhibitor molecule from the DFT calculations was found to be −5.069 and −5.429, respectively, which is in support of the protonated counterpart. Figures 14 and 15 indicate that the HOMO and LUMO are predominantly distributed over the −NO_2_ substituted benzene ring, pyrazolone ring, imine group, and hetero atoms, making them active centers in the inhibitor molecule for the adsorption on the steel surface.^20^

The obtained quantum chemical parameters of neutral and protonated species of the inhibitor are not similar, and they differ significantly because of the difference in electron density distribution between neutral and protonated inhibitor molecule. Besides, the values of *E*_HOMO_ and *E*_LUMO_ in the case of the protonated molecule imply its interaction with the metal surface via electron donation to the unoccupied d orbital of the metal and/or by accepting electrons easily from the same. Generally, a high value of dipole moment (μ) and small value of energy gap (Δ*E*) favor the strong interaction of the inhibitor molecule on the metal surface, which is true in the case of the protonated counterpart of 4-NBAAP. In addition, a higher dipole moment w.r.t. the protonated inhibitor is ascribed to dominant dipole–dipole interactions (Table 6).^21^

**Table 6:** Quantum Chemical Parameters of 4-NBAAP

quantum chemical parameters	neutral species of 4-NBAAP molecule	protonated species of 4-NBAAP molecule
*E*_HOMO_ (eV)	–5.78	–4.43
*E*_LUMO_ (eV)	–2.83	–6.15
Δ*E* (eV)	2.95	–1.72
dipole moment μ (Debye)	9.09	11.65
total energy (kcal/mol)	–711,464	–711,839

### 3.6 Mechanism of Inhibition

From potentiodynamic polarization results, it is affirmed that the molecule behaves as a mixed type of inhibitor. Corrosion inhibition is very much dependent on the extent to which the inhibitor adsorbs on the metal surface, and this adsorption depends on the structure of the inhibitor, the nature and charge of the metal, and the type of corrosive medium. The corrosive medium (inhibitor completely dissolved in aqueous 1 M HCl solution, in the current study), in turn the solvent, plays a crucial role in achieving higher effectiveness of the inhibitor chosen. It has been claimed that the solvents function by decreasing the viscosity and ensuring the inhibitor formulation’s stability in different environments. In addition, the solvent also is intended to improve the solubility, dispersibility, especially in acids, and wettability on the acid–steel interface.^59,60^

As 4-NBAAP possesses various active centers such as the imine group, –NO_2_ substituted benzene ring, pyrazolone ring, etc., the adsorption phenomenon becomes complex to understand, and the following mechanism has been proposed. In an acidic medium, 4-NBAAP might be protonated at the N atom of the imine group, becoming a cation that exists in equilibrium with the corresponding neutral counterpart. The same can be represented as follows
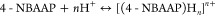


Therefore, it is considered that in an acidic solution, the inhibitor exists as the protonated species and as a neutral molecule. The cationic form of the inhibitor 4-NBAAP is likely to adsorb directly at the cathodic sites, minimizing the hydrogen evolution reaction. At the corrosion potential, MS attains a positive charge when exposed to an acidic medium. This causes chloride ions from the medium to adsorb on the metal surface, leading to aggregation of negative charges toward the solution, favoring the adsorption of the protonated inhibitor onto the steel surface driven by electrostatic attraction (physisorption), controlling the anodic reaction, i.e., metal dissolution.^20,21^ On the other hand, neutral 4-NBAAP may also adsorb at a positively charged steel surface via chemisorption by sharing electrons of electronegative atoms such as N and O and also by donor–acceptor interactions between π-electrons of antipyrine and benzaldehyde rings and the vacant d orbital of iron.^20,21^ On the other hand, the protonated inhibitor molecule may also expected to be adsorbed directly on the cathodic sites in completion with hydrogen ions, thereby minimizing hydrogen evolution, the cathodic reaction. So, these interactions between the steel surface and the inhibitor molecule result in good adsorption, inhibiting the corrosion to a higher extent, which proves 4-NBAAP to be an excellent corrosion inhibitor in an aggressive HCl medium.

## 4 Conclusions

Results show that 4-NBAAP effectively inhibits MS corrosion in 1 M hydrochloric acid. By inhibiting both anodic and cathodic reactions, it functions as a mixed-type inhibitor. The rate of corrosion increases with temperature and decreases with the inhibitor concentration. The spontaneous and exothermic nature of 4-NBAAP adsorption is modeled by the Langmuir adsorption isotherm. Thermodynamic and kinetic parameters of the adsorption and corrosion processes are established. The trends obtained from the measurements of weight loss experiments are comparable to those of the electrochemical results. The variation in the surface morphology of metal samples in the presence and absence of the inhibitor molecule also pronounces the excellent protection ability of 4-NBAAP. Quantum chemical studies pronounced all our experimental conclusions to be in line and assisted in understanding the mechanism of corrosion inhibition.

## Supporting Information Available

Mass, UV, FTIR, ^1^H NMR, ^13^C NMR spectral data; corrosion parameters in different inhibitor concentrations at different temperatures; weight loss experimentation; and images of synthesized crystals of the inhibitor (PDF)
