# Supplementary material for: Quantum Chemical and Experimental Evaluation of a 4-Amino-Antipyrine Based Schiff Base as Corrosion Inhibitor for Steel Material
Source: ACS Omega. 2024 Mar 8;9(11):13262–73. doi: 10.1021/acsomega.3c10048 (PMC10956091; doi:10.1021/acsomega.3c10048)
Supplement: Supplementary file 1 — ao3c10048_si_001.pdf [file ao3c10048_si_001.pdf]

SUPPLEMENTARY FILE

Evaluation of a 4-amino-antipyrine based Schiff base as corrosion inhibitor for steel material

Ashwini Narayanswamy[1], Dileep Ramakrishna[1], P.V. Raja Shekar[2], Shashanka Rajendrachari[3*], Ranganatha Sudhakar[1*]

[1]Department of Chemistry, Presidency University, Rajanakunte, Bengaluru 560064, India
[2]Department of Physics, SR University, Warangal 506371, India
[3]Department of Metallurgical and Materials Engineering, Bartin University, Bartin-74100, Turkey

***Corresponding authors:** kamath.ranganath@gmail.com, shashankaic@gmail.com

Contents

Materials and Methods

Figure S1: Mass Spectrum of complexes 4-[(4-Nitrobenzylidene)-amino]-antipyrine (4-N BAAP).

Figure S2: UV Spectrum of 4-[(4-Nitrobenzylidene)-amino]-antipyrine (4-NBAAP).

Figure S3: FTIR Spectrum of 4-[(4-Nitrobenzylidene)-amino]-antipyrine (4-NBAAP).

Figure S4: ^{1}H NMR Spectrum of 4-[(4-Nitrobenzylidene)-amino]-antipyrine (4-NBAAP).

Figure S5: ^{13}C NMR Spectrum of 4-[(4-Nitrobenzylidene)-amino]-antipyrine (4-NBAAP).

Figure S6: Corrosion parameters of mild steel in 1M HCl in different inhibitor concentration at different temperatures.

Figure S7: Weight loss measurement process and fresh crystals of 4-[(4-Nitrobenzylidene)-amino]-antipyrine (4-NBAAP).

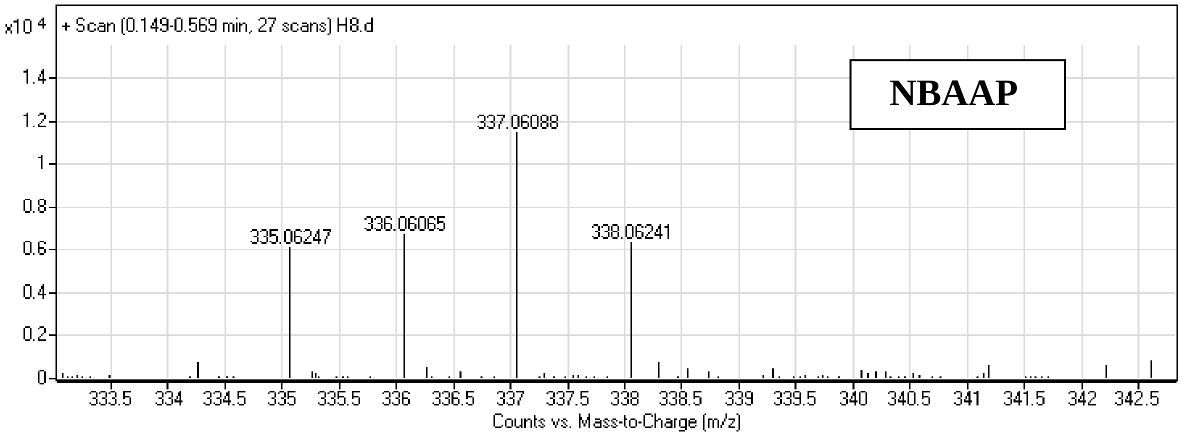

Figure S1: Mass Spectrum of complexes 4-[(4-Nitrobenzylidene)-amino]-antipyrine (4-NBAAP).

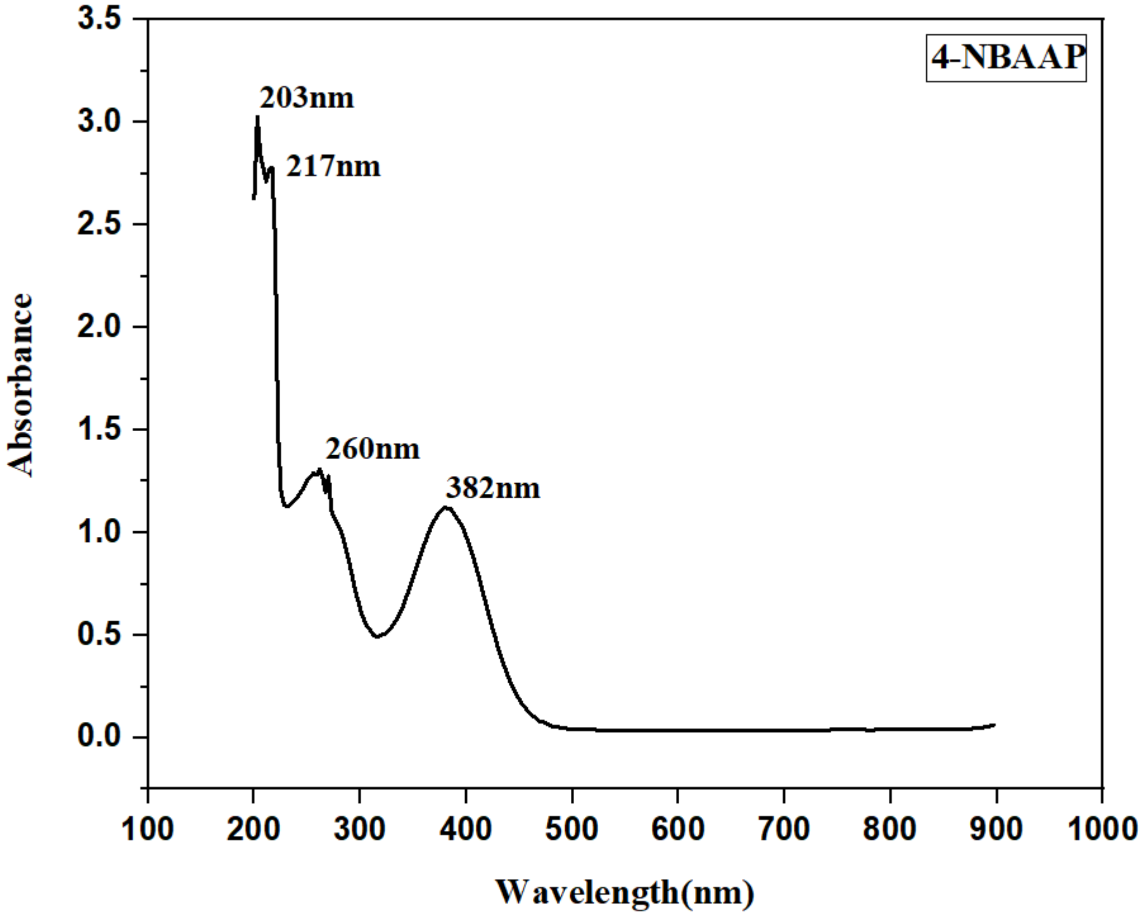

Figure S2: UV Spectrum of 4-[(4-Nitrobenzylidene)-amino]-antipyrine (4-NBAAP).

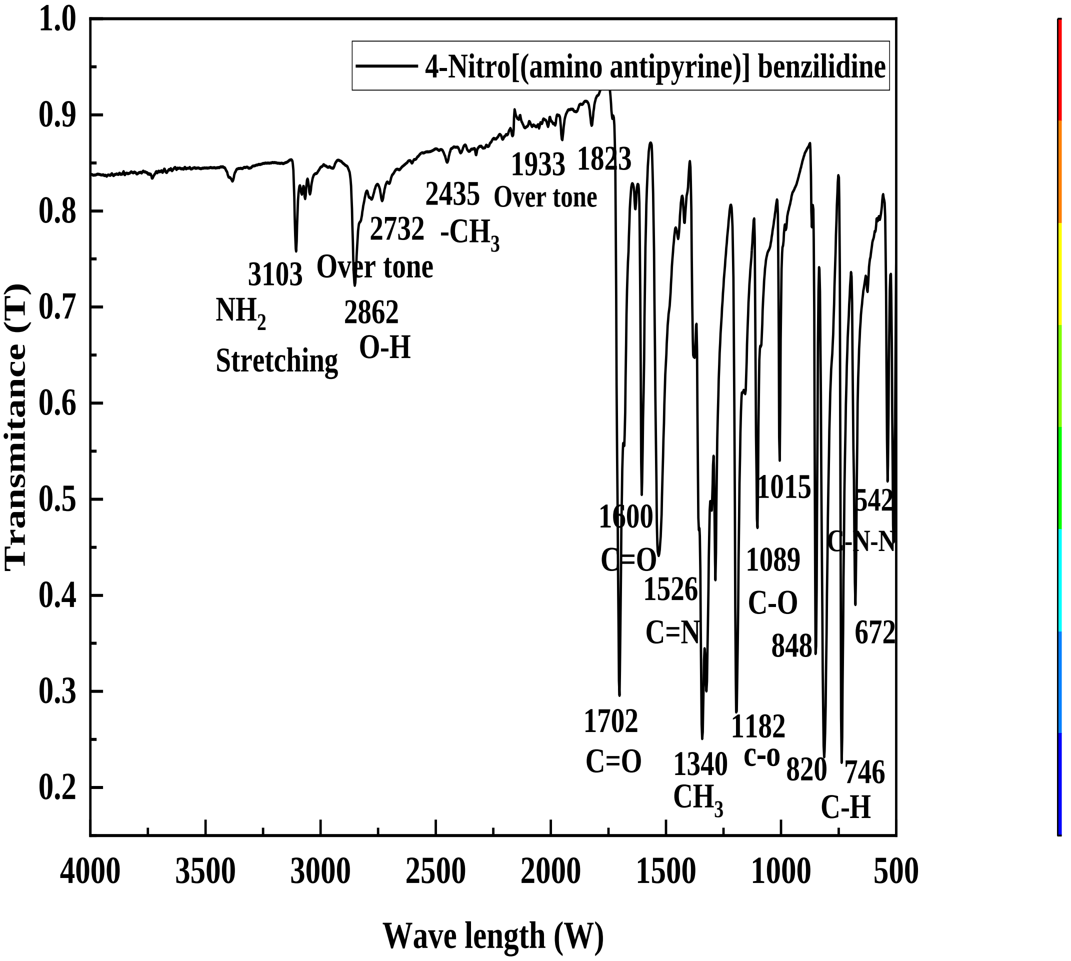

Figure S3: FTIR Spectrum of 4-[(4-Nitrobenzylidene)-amino]-antipyrine (4-NBAAP)

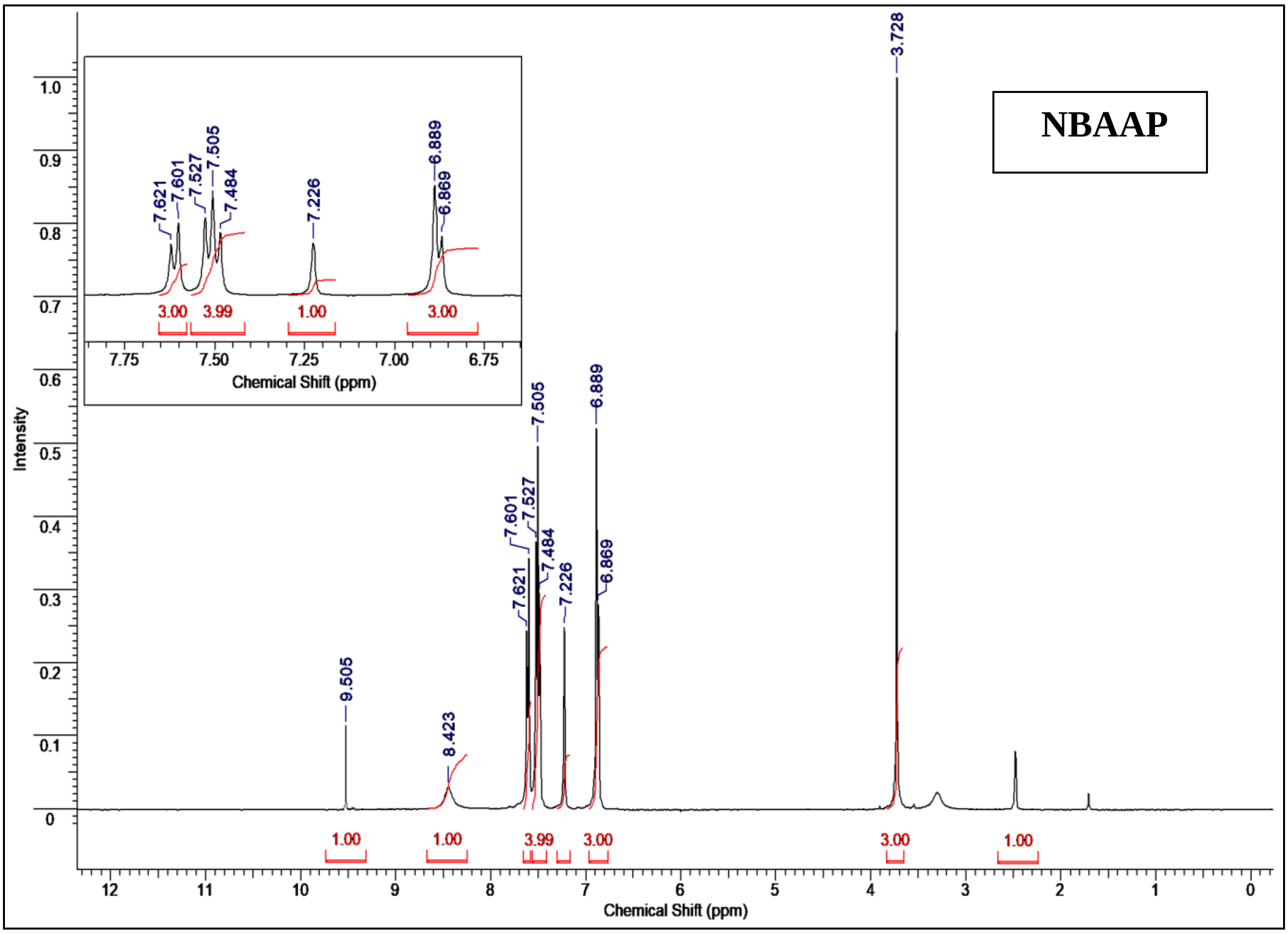

Figure S4: ^{1}H NMR Spectrum of 4-[(4-Nitrobenzylidene)-amino]-antipyrine (4-NBAAP).

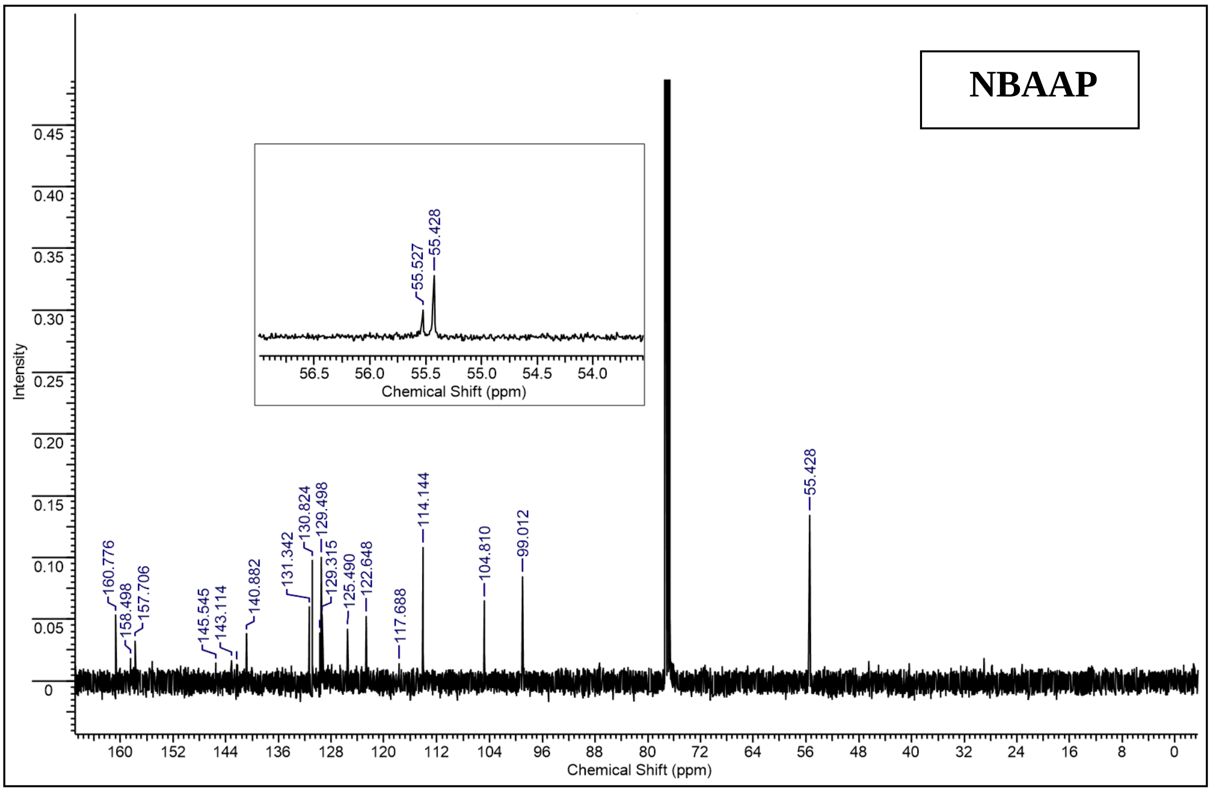

Figure S5: ^{13}C NMR Spectrum of 4-[(4-Nitrobenzylidene)-amino]-antipyrine (4-NBAAP)

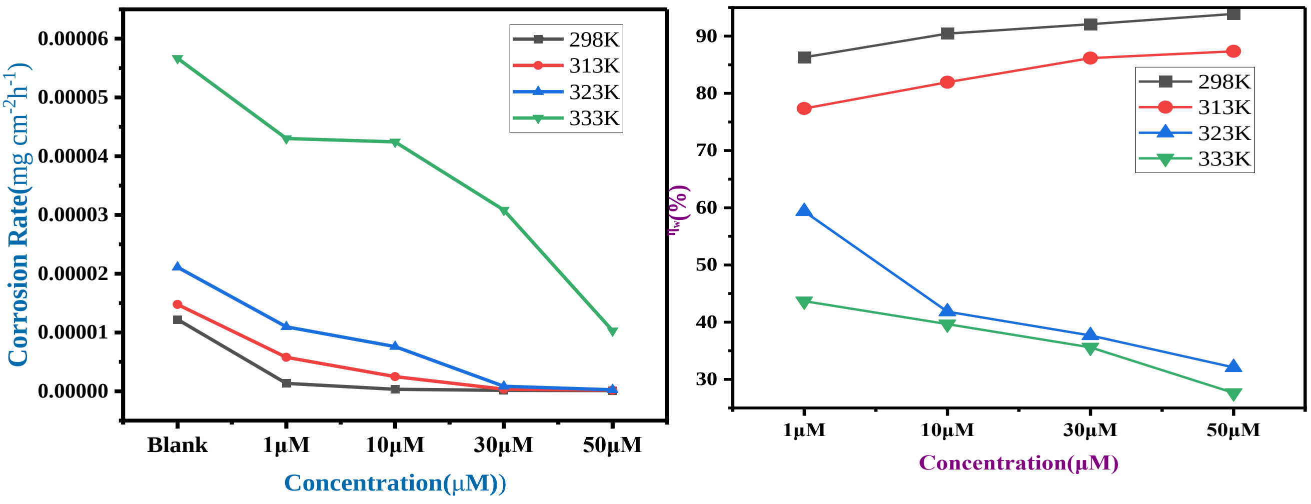

Figure S6: Corrosion parameters of mild steel in 1M HCl in different inhibitor concentration at different temperatures.

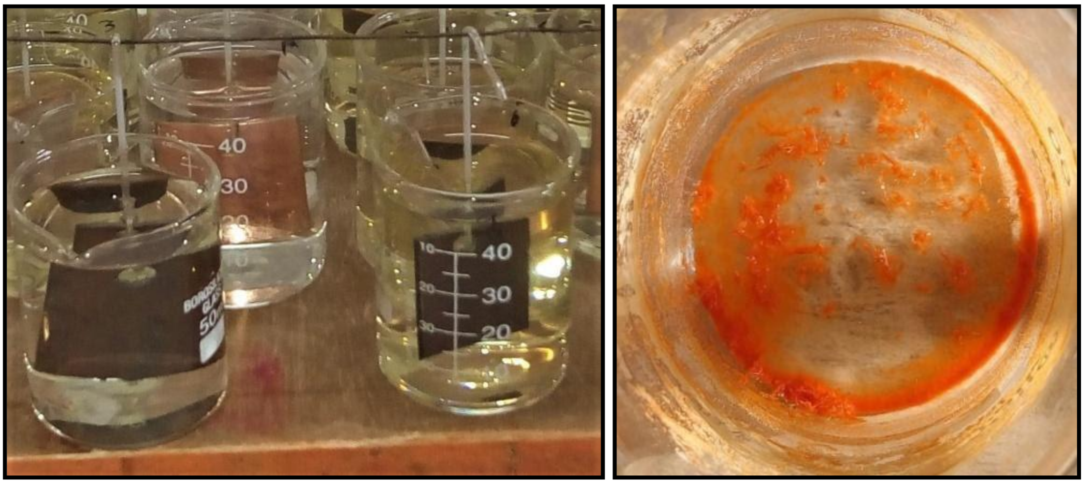

Figure S7: Weight loss measurement process and fresh crystals of 4-[(4-Nitrobenzylidene)-amino]-antipyrine (4-NBAAP).
